# Identifying potential biomarkers and molecular mechanisms related to arachidonic acid metabolism in vitiligo

**DOI:** 10.3389/fmolb.2025.1536477

**Published:** 2025-02-26

**Authors:** Xiaoqing Li, Li Yang, Longfei Zhu, Jingying Sun, Cuixiang Xu, Lijun Sun

**Affiliations:** ^1^ Shaanxi Provincial Key Laboratory of Infection and Immune Diseases, Shaanxi Provincial People’s Hospital, Xi’an, Shaanxi, China; ^2^ Department of Dermatology, Shaanxi Provincial People’s Hospital, Xi’an, Shaanxi, China; ^3^ Department of Dermatology, The Second Affiliated Hospital of Xi’an Jiaotong University, Xi’an, Shaanxi, China; ^4^ Shaanxi Province Research Center of Cell Immunological Engineering and Technology, Shaanxi Provincial People’s Hospital, Xi’an, Shaanxi, China

**Keywords:** vitiligo, arachidonic acid metabolism, key genes, machine learning, biomarker

## Abstract

**Background:**

Numerous studies have reported that dysregulation of fatty acid metabolic pathways is associated with the pathogenesis of vitiligo, in which arachidonic acid metabolism (AAM) plays an important role. However, the molecular mechanisms of AAM in the pathogenesis of vitiligo have not been clarified. Therefore, we aimed to identify the biomarkers and molecular mechanisms associated with AAM in vitiligo using bioinformatics methods.

**Methods:**

The GSE75819 and GSE65127 datasets were used in this study as the training and validation sets, respectively, along with 58 AAM-related genes (AAM-RGs). The differentially expressed genes (DEGs) between the lesional and control groups in the training set were identified through differential expression analysis. A biomarker-based nomogram was constructed to predict the risk of vitiligo.

**Results:**

15 overlapping candidate genes were obtained between the DEGs and AAM-RGs. Machine-learning algorithms were used to identify six key genes as *PTGDS*, *PNPLA8*, *FAAH*, *ABHD12*, *PTGS1*, and *MGLL*. In both the training and validation sets, *PTGDS*, *PNPLA8*, and *MGLL*. In both the training and validation sets, *PTGDS*, *PNPLA8*, and *MGLL* were regarded as biomarkers. A nomogram based on these biomarkers showed potential for predicting the risk of vitiligo. Functional enrichment, immune cell infiltration, and regulatory network analyses were used to elucidate the molecular mechanisms.

**Conclusion:**

In conclusion, *PTGDS*, *PNPLA8*, and *MGLL* were implicated in AAM to influence the pathogenesis of vitiligo. These findings offer insights into vitiligo treatment, although further research is needed for a comprehensive understanding.

## 1 Introduction

Vitiligo is a localized or generalized depigmentation skin disorder characterized by decreased color, increased whiteness, and distinct boundaries (Lyu and Sun, 2022). The pathogenesis of vitiligo is unclear and may be related to factors such as immune dysfunction, oxidative stress, melanocyte dysfunction, and intestinal flora disturbance (Post et al., 2023). At present, there are very few treatment methods for vitiligo and different degrees of limitations that do not meet the demands of patients (Cunningham and Rosmarin, 2023). Therefore, further exploration of safer, more efficient, and inexpensive treatment strategies is an urgent problem in vitiligo research.

Polyunsaturated fatty acids are essential substances needed for maintaining human health and mainly include the ω-3 and ω-6 types (Duan et al., 2023). Arachidonic acid (AA) is an essential fatty acid in the human body belonging to the ω-6 type of polyunsaturated fatty acids. AA and its metabolites have important biological activities in inflammatory responses, immune regulation, and signal transmission, which are involved in the occurrence and development of various diseases (Zhang et al., 2023). Growing evidence suggests that arachidonic acid metabolism (AAM) is closely associated with autoimmune diseases, such as psoriasis, systemic lupus erythematosus, and rheumatoid arthritis (Chaaba et al., 2023; Tu et al., 2023; Ye et al., 2021; Turolo et al., 2021). Moreover, it has been reported that the incidence of metabolic syndrome in patients with vitiligo is significantly higher than in healthy controls (Liang et al., 2022). Studies have shown that dysregulation of the fatty acid metabolic pathways is associated with the pathogenesis of vitiligo (Ni et al., 2020; Ye et al., 2022). However, there are no relevant studies on how AAM participates in the occurrence and development of vitiligo. Therefore, in-depth exploration of vitiligo based on metabolic disorders is needed.

In the present study, the weighted gene coexpression network analysis (WGCNA) tool and machine learning were used to explore the key genes related to AAM in vitiligo. Based on bioinformatics analysis of the biological pathways and immune microenvironments of the key genes, the molecular regulatory mechanisms of these genes were further explored to provide a new reference for the clinical diagnosis and treatment of vitiligo.

## 2 Materials and methods

### 2.1 Data source

The GSE75819 dataset (platform: GPL6884), which includes 15 lesions and 15 control skin tissues from vitiligo patients (Supplementary Table S1), was downloaded from the Gene Expression Omnibus (GEO) database (https://www.ncbi.nlm.nih.gov/geo/) for use as the training set. The validation set GSE65127 (platform: GPL570) contained 10 lesions and 20 control skin tissue samples. A total of 58 AAM-related genes (AAM-RGs) were also downloaded from MSigDB (https://www.gsea-msigdb.org/gsea/msigdb).

### 2.2 Identification and functional enrichment analysis of the candidate genes

The limma package (v3.54.0) (Ritchie et al., 2015) was used to analyze the differentially expressed genes (DEGs) of the lesional and control groups from the training set with |log_2_FC|>0.5 and adj. *P* < 0.05. The volcano plot was used to visualize the DEGs using the ggplot2 package (v3.4.1). The top-10 upregulated and downregulated DEGs were labeled in the volcano plot, and their expression heatmap was obtained using the circlize package (v0.4.15) (Gu et al., 2014). Furthermore, the intersection between the DEGs and AAM-RGs was obtained to identify the candidate genes. To probe the functions of these candidate genes, the gene ontology (GO) and Kyoto Encyclopedia Of Genes And Genomes (KEGG) enrichment analyses were conducted using the clusterProfiler package (v4.2.2) (Wu et al., 2021) with a threshold of adj. *P* < 0.05. To further explore the relationships between the proteins encoded by the candidate genes and understand their potential functions, the protein–protein interaction (PPI) network was constructed based on the protein interaction information in the STRING database by setting a medium confidence level >0.4.

### 2.3 Biomarker screening

The least absolute shrinkage and selection operator (LASSO) and support vector machine recursive feature elimination (SVM-RFE) algorithms were employed on the training set to screen the feature genes based on the candidate genes. LASSO regression analysis was then performed using the glmnet package (v4.1-4) (Friedman et al., 2010), where the optimal lambda value (Lambda.min) was obtained through 10-fold cross-validation to minimize the model error rate. At this stage, the feature genes 1 whose regression coefficients were not penalized to 0 were selected. Meanwhile, the SVM-RFE was implemented using the caret package (v6.0-93, https://cran.r-project.org/web/packages/caret/index.html) to obtain feature genes 2. Subsequently, the feature genes from the two machine learning algorithms were combined to yield the key genes. The expression levels of these key genes in the lesional and control groups from the training and validation sets were analyzed separately, and those with significant expression differences and consistent trends in both datasets were considered biomarkers for further analyses. To explore the performances of the biomarkers in differentiating vitiligo samples from the control samples, the “pROC” package (v1.18.0) was used on the training set GSE75819 to draw the receiver operating characteristics (ROC) curves of the candidate key genes and to calculate the area under the curve (AUC). An AUC value greater than 0.7 indicated that the corresponding gene had relatively good diagnostic performance.

### 2.4 Construction and verification of the nomogram

To predict the risk of vitiligo, the rms package (https://CRAN.R-project.org/package=rms) was applied to create a nomogram based on the biomarkers identified from the training set with lesional status as the outcome. Then, the predictive ability of the nomogram was evaluated using the calibration curve, decision curve analysis (DCA), and clinical impact curves (CICs).

### 2.5 Gene set enrichment analysis (GSEA)

To investigate the biological pathways involved with the biomarkers, GSEA was performed on the biomarkers from the training set using the clusterProfiler package, and the Spearman correlation coefficient of each biomarker with all the other genes was computed using “c2cp.kegg.v7.5.1.symbols.gmt” as the reference set of genes. The genes were then sorted in descending order to produce a list of related genes corresponding to each biomarker for the GSEA.

### 2.6 Immune cell infiltration analysis

We used the GSVA package (v1.42.0) (Hänzelmann et al., 2013) to compute the enrichment scores of 28 immune infiltrating cells for all samples in the training set based on the ssGSEA algorithm. Then, these enrichment scores were displayed in a box plot to identify the differential immune cells between the lesional and control groups. For the training set, the correlations between the differential immune infiltrating cells were analyzed using the corrplot package (v0.92), and the relationships between the biomarkers and differential immune cells were assessed by the Spearman correlation analysis to explore the immune microenvironment characteristics specific to vitiligo.

### 2.7 Chromosome localizations and regulatory networks of the biomarkers

The distribution of biomarkers on the chromosomes was initially visualized using Circos (http://circos.ca/) with the training set. The GeneMania network was then constructed (http://www.genemania.org/) to predict other genes related to the biomarker functions and biological pathways. The transcription factors (TFs) regulating the biomarkers were retrieved from the Network Analyst database (https://www.networkanalyst.ca/), and the upstream miRNAs of the biomarkers were predicted for the training set using the ENCORI and miRWalk databases. Then, the miRNAs from the two databases were overlapped to obtain the intersecting miRNAs. Finally, the miRNA–mRNA–TF regulatory network was constructed using Cytoscape software.

### 2.8 RT-PCR

Skin tissues from vitiligo patients (generally from the lower back) who were diagnosed as having advanced non-segmental vitiligo, including two men and two women aged 19–52 years, were obtained from the Department of Dermatology of Shaanxi Provincial People’s Hospital. The exclusion criteria were as follows: participants had other related skin or autoimmune diseases in the past 6 months or had received systemic therapy with glucocorticoids and immunosuppressive drugs. The skin tissues of healthy people were obtained from the plastic surgery department of Shaanxi Provincial People’s Hospital; these people had no genetic history, no systemic immune diseases, and no acute or chronic history and included two men and two women aged 21–56 years. This study was conducted in accordance with the guidelines of the Declaration of Helsinki. The Ethics Committee of Shaanxi Provincial People’s Hospital approved the study, and all patients provided written informed consent. Total RNA was extracted from the tissue samples using the Total RNA kit (Omega Bio-tec, Inc.). Then, the total RNA was transcribed into cDNA using the PrimeScript™ RT kit (TaKaRa, Dalian, China). RT-PCR was then performed using the PCR Master Mix (Thermo Fisher). The primers were designed using primer premier 5.0 software, and the sequences were as follows: PNPLA8 forward: 5′-AGCCTACAAGTCCTTCTGCGATAC-3′; PNPLA8 reverse: 5′-TCCGTGGGACGAGAAAGAAAGTTAG-3′; PTGDS forward: 5′-GGTCTCCGTGCAGCCCAAC-3′; PTGDS reverse: 5′-TGGACAACGCCGCCTTCTTC-3′; MGLL forward: 5′-TCCAACTGCTGAATGCCGTCTC-3′; MGLL reverse: 5′-TTGTCCTGGCTCTTGGCTAACTC-3′; β-actin forward: 5′-CTGGAACGGTGAAGGTGACA-3′; β-actin reverse: 5′-AAGGGACTTCCTGTAACAATGCA-3′. The PCR thermocycling conditions consisted of an initial 5 min of denaturation at 95°C, followed by 30 cycles at 95°C for 30 s, 54°C for 30 s, and 72°C for 40 s. The change in transcript abundance was calculated using the 2^−ΔΔCT^ method.

### 2.9 Statistical analysis

R software (v4.2.2) was used for the statistical analyses. The differences between groups were analyzed by Wilcoxon’s test, and *P* < 0.05 was set as the statistical significance.

## 3 Results

### 3.1 Identification and enrichment analyses of the candidate genes

**FIGURE 1:**
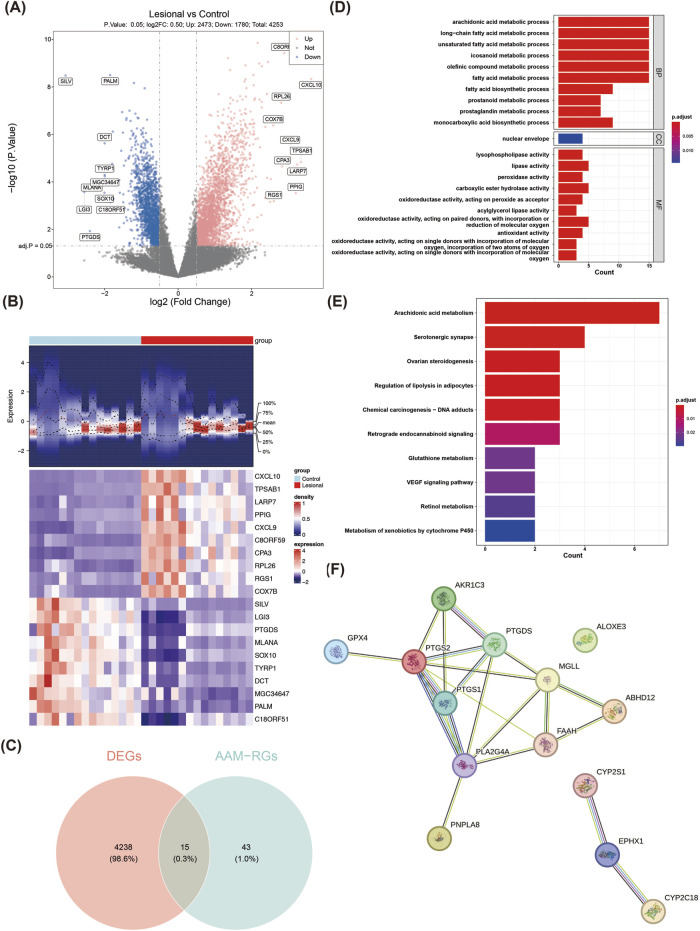
Identification and enrichment analyses of the candidate genes in normal and vitiligo samples. **(A)** Volcano plot of the differentially expressed genes (DEGs). The red dots represent the significantly upregulated genes, and the blue dots represent the significantly downregulated genes. **(B)** Heatmap of the DEGs. The top part is a density heatmap of the expression levels of the upregulated and downregulated genes in the samples showing the lines of the quartile means; the lower part is the expression heatmap of the top-10 upregulated and downregulated genes in the samples. Each square represented a sample, with red representing upregulation and purple representing downregulation. **(C)** Venn diagram of the interacting targets. **(D)** GO enrichment analysis results. **(E)** KEGG pathway enrichment analysis results. **(F)** PPI network of the candidate genes.

A total of 4,253 DEGs (2,473 upregulated and 1,780 downregulated) were obtained from the lesional and control groups of the training set (Figure 1A). The expressions of the top-10 upregulated and downregulated DEGs in the two groups are shown as a heatmap in Figure 1B. By overlapping the 4,253 DEGs with the 58 AAM-RGs, 15 candidate genes were obtained for subsequent analyses (Figure 1C). These 15 candidate genes were enriched in 221 GO items (e.g., AAM process, long-chain fatty acid metabolic process, and unsaturated fatty acid metabolic process) (Figure 1D; Supplementary Table S2) and 52 KEGG pathways (e.g., AAM, glutathione metabolism, and VEGF signaling pathway) (Figure 1E; Supplementary Table S3). The enrichment analysis results showed that the DEGs were widely involved in the AAM pathway associated with the synthesis of lipid molecules such as prostaglandins and leukotrienes that play key roles in various physiological processes, including inflammation and immunity. Furthermore, the PPI network showed 21 interactions between 14 proteins (GPX4-PTGS2, MGLL-FAAH, PTGS1-PTGDS etc.), of which ALOXE3 was an isolated target with no protein interactions with the other genes (Figure 1F).

### 3.2 Screening of biomarkers

**FIGURE 2:**
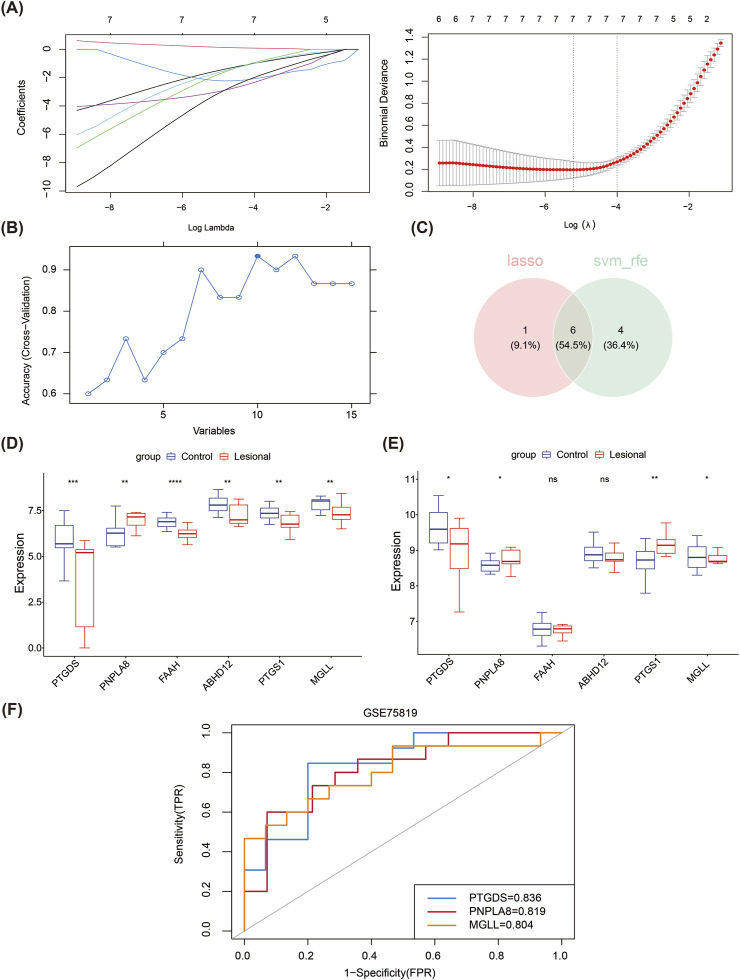
Machine learning approach for screening biomarkers. **(A)** LASSO logistic regression to screen candidate genes. **(B)** SVM-RFE for screening the candidate genes. **(C)** Venn diagrams of the hub genes between LASSO and SVM-RFE. **(D)** Expressions of the six hub genes in the training dataset. **(E)** Expressions of the six hub genes in the validation dataset. **(F)** The receiver operating characteristic curves of the three identified biomarkers show that the area under each curve is >0.7, as indicated in the inset legend.

Based on the 15 candidate genes identified from the training set, six key genes (*PTGDS*, *PNPLA8*, *FAAH*, *ABHD12*, *PTGS1*, and *MGLL*) were screened through intersection with seven feature genes 1 produced by the LASSO algorithm (lambda.min = 0.005445) and 10 feature genes 2 produced by the SVM-RFE algorithm (Figures 2A–C). In the training set, *PNPLA8* was found to be significantly overexpressed in the lesion group, while the other key genes were significantly underexpressed (Figure 2D). In the validation set, *PNPLA8* and *PTGS1* were significantly overexpressed, while *PTGDS* and *MGLL* were significantly underexpressed in the lesion group (Figure 2E). *FAAH* and *ABHD12* showed no significant differences between the lesional and control groups. Therefore, *PTGDS*, *PNPLA8*, and *MGLL* were selected as biomarkers. The subsequent ROC results showed that the AUC values of *PTGDS*, *PNPLA8*, and *MGLL* were all greater than 0.7, indicating that these biomarkers had good diagnostic performances (Figure 2F).

### 3.3 Prediction of vitiligo from nomogram modeling

**FIGURE 3:**
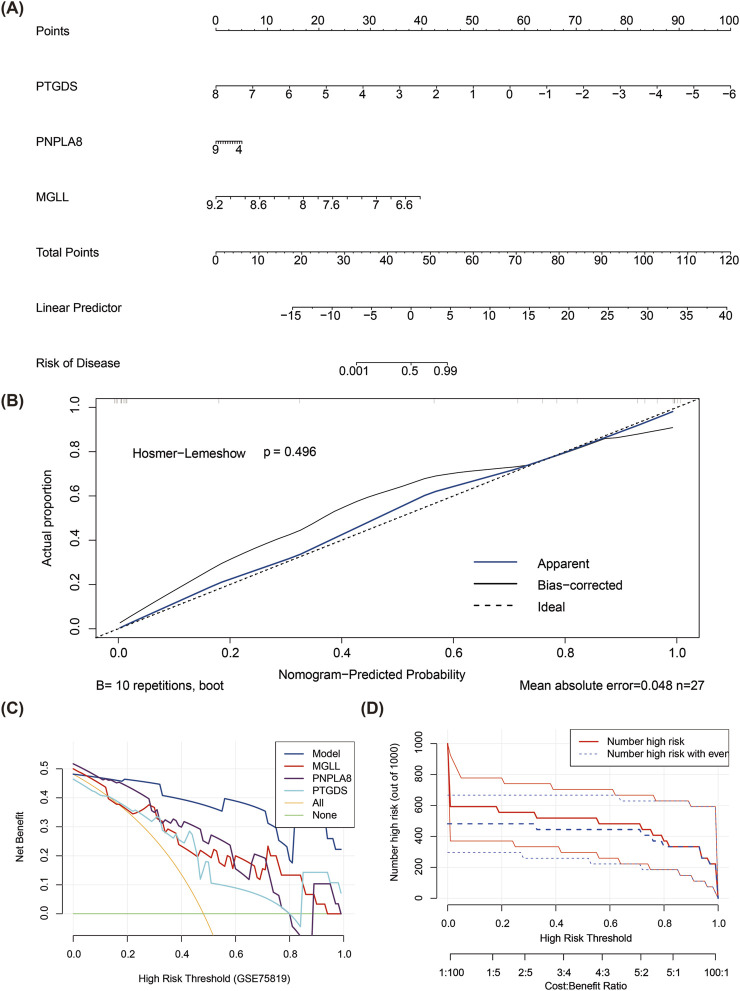
Construction of nomogram model for vitiligo prediction. **(A)** Nomogram model of PTGDS, MGLL, and PNPLA8. **(B)** Calibration curve for evaluating the predictive ability of the column chart mode. **(C)** Decision curve analysis for evaluating the predictive ability of the column chart model. **(D)** Clinical impact curve for evaluating the predictive ability of the column chart model.

The total points were computed based on the expressions of the three biomarkers to create a nomogram model for vitiligo prediction (Figure 3A). In the calibration curve for the nomogram, the *P* value of the Hosmer–Lemeshow test was greater than 0.05 (*P* = 0.496), indicating that there was no notable difference between the predicted and true values and that the model had a high predictive accuracy (Figure 3B). The DCA suggested that the net benefit of the nomogram model was higher than the extreme curves (Figure 3C). The CICs also indicated that the model might have high clinical validity (Figure 3D).

### 3.4 GSEA

**FIGURE 4:**
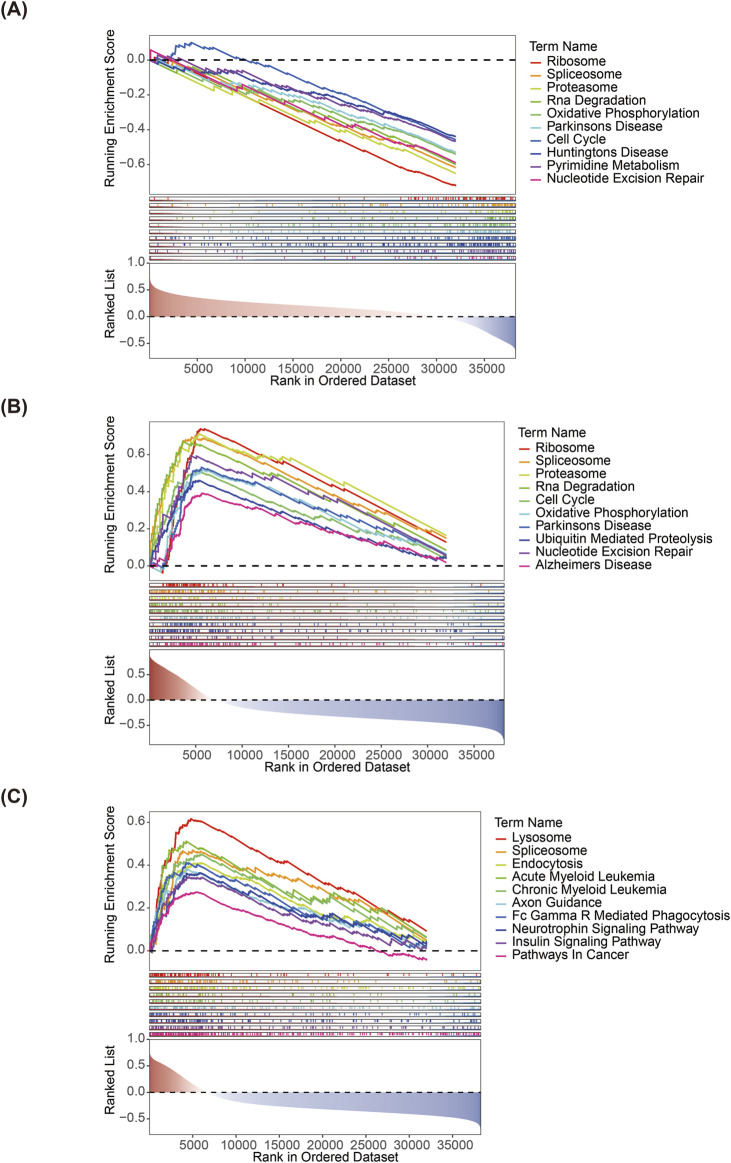
Gene set enrichment analyses of **(A)** *PTGDS*, **(B)** *PNPLA8*, and **(C)** *MGLL*.

According to the GSEA, *PTGDS* and *PNPLA8* were significantly enriched in the ribosome, proteasome, cell cycle, and other pathways; *MGLL* was significantly enriched in the lysosome, endocytosis, Fc gamma R mediated phagocytosis, and other pathways (Figures 4A–C).

### 3.5 Immune infiltration analysis

**FIGURE 5:**
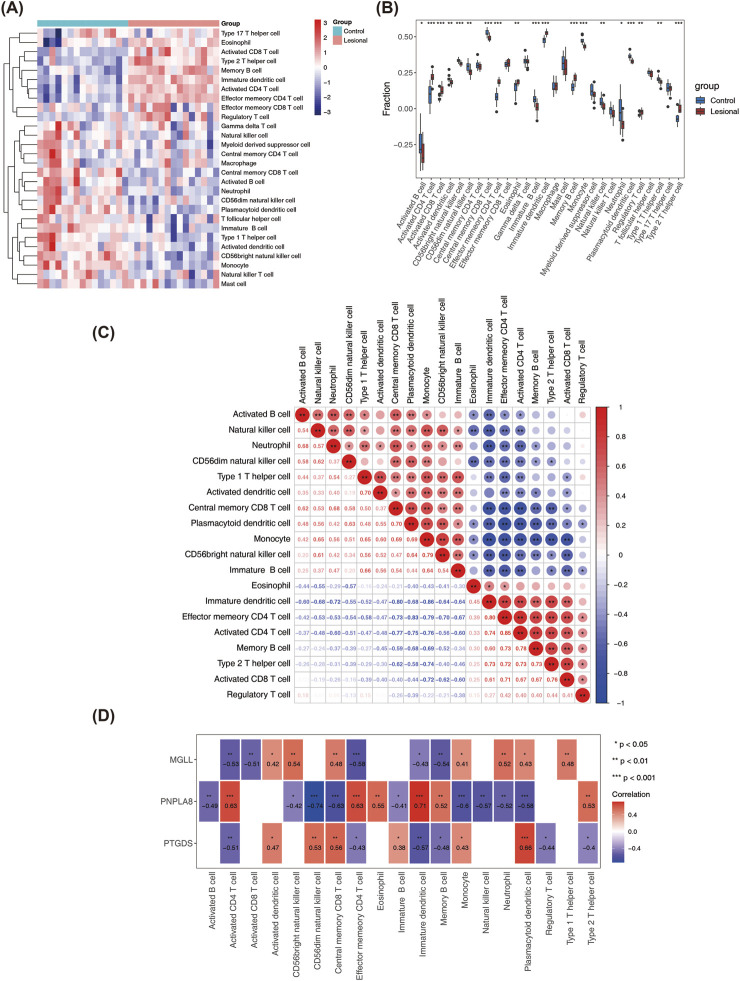
Analysis of immune infiltration. **(A)** Heatmap of the distribution of immune infiltrating cells between lesional and control samples. **(B)** Box plot of the 28 immune cell expressions between the sample groups. **(C)** Heatmap of correlations between the immune cells. **(D)** Analysis of correlations between the three key genes and distinct immune cells. (*P < 0.05, **P < 0.01, ***P < 0.001).

Immune infiltration analysis was performed on 28 immune cells and three biomarkers. Initially, the heatmap showed the distribution of the enrichment scores of the 28 immune infiltrating cells between the lesion and control samples (Figure 5A). It was noted that the enrichment scores of 19 immune infiltrating cells, such as activated CD8 T cells, activated CD4 T cells, and effector memory CD4 T cells, were significantly different between the two groups (*P* < 0.05) (Figure 5B). Figure 5C shows the correlations between the 19 differential immune cells. There was an extremely significant negative correlation between monocytes and immature dendritic cells (cor = −0.86), while there was an extremely significant positive correlation between the activated CD4 T cells and effector memory CD4 T cells (cor = 0.85). The relationships between the immune cells and biomarkers showed that *PTGDS* and *MGLL* were both significantly negatively correlated with immune cells such as activated CD4 T cells, effector memory CD4 T cells, immature dendritic cells, and memory B cells as well as significantly positively correlated with central memory CD8 T cells, monocytes, plasmacytoid dendritic cells, and other cells. However, *PNPLA8* had the opposite correlations as the above two genes, suggesting that *PNPLA8* might play an opposite role to those of *PTGDS* and *MGLL* in the regulation of immune cells (Figure 5D).

### 3.6 Construction of the TF–miRNA–mRNA network

**FIGURE 6:**
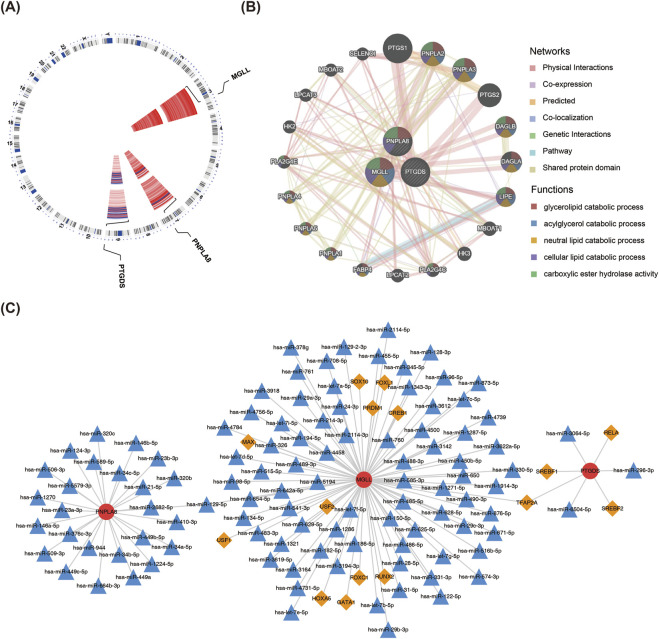
TF–miRNA–mRNA regulatory network. **(A)** Chromosome localization analysis of the three key genes. **(B)** GeneMania analysis of the key genes. **(C)** Analysis of the transcription factors and miRNAs regulatory network of the key genes. The red circles are the key genes, orange diamonds are the transcription factors, and blue triangles are the miRNAs.

The chromosomal localization analysis revealed that *PTGDS* was located on chromosome 9, *PNPLA8* on chromosome 7, and *MGLL* on chromosome 3 (Figure 6A). A total of 20 genes related to biomarker functions were analyzed using GeneMania, and the functions of these genes were mainly related to lipid metabolic processes (Figure 6B). The results of TF predictions showed that 11 TFs could regulate the expression of *MGLL* while 4 TFs could regulate the expression of *PTGDS*. Among these TFs, TFAP2A was identified as a regulator of both biomarkers. In addition, the TF-miRNA-mRNA network showed the intersecting miRNAs and TFs corresponding to each of the three biomarkers (Figure 6C).

### 3.7 Target gene expression verifications in clinical samples

**FIGURE 7:**
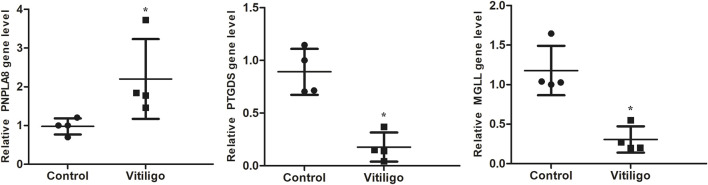
RT-PCR validation of the identified biomarkers. Skin tissues of four patients with vitiligo and four normal controls were collected for RT-PCR. (*P < 0.05).

The RT-PCR results showed that compared with the control group, the expressions of *PTGDS* and *MGLL* in the skin tissues of vitiligo patients were significantly downregulated, whereas the expression of *PNPLA8* was significantly upregulated (Figure 7). These results were consistent with the findings of the bioinformatics analysis, confirming that the target genes may play crucial roles in vitiligo.

## 4 Discussion

Vitiligo is a depigmentation skin disorder caused by many factors, including autoimmune, metabolic, or their combination. The incidence of metabolic syndrome was reported to be significantly higher in patients with vitiligo than healthy controls (Tanacan and Atakan, 2020). The metabolic pathways of fatty acids, including arachidonic acid, are significantly associated with the development of vitiligo (Ni et al., 2020), but their action mechanisms in vitiligo have not been studied.

Machine learning algorithms are widely used in bioinformatics and genomics. In the present study, LASSO and SVM-RFE algorithms were used to screen the key genes related to AAM in vitiligo. The genes selected by LASSO focus on linear relationships, while the genes selected by SVM-RFE focus on classification performance. Combining the two algorithms can thus reduce the risk of overfitting due to the limitations of a single algorithm (Liang et al., 2022). The selected key genes were not only based on linear relationships and classification performance but also more representative and stable. In terms of biomarker prediction, this key gene set can more accurately reflect the characteristics of the biomarkers and improve prediction accuracy.

In this study, we first obtained six AAM-RGs in vitiligo by searching the transcriptome dataset in the GEO database along with difference analysis and machine learning; then, *PTGDS*, *PNPLA8*, and *MGLL* were verified as the three key genes through the validation set. Prostaglandin D synthase (PTGDS) is an important enzyme in the AAM pathway that is responsible for the synthesis of prostaglandin D2 (PGD2) and plays a critical role in the transportation of fat-soluble substances (Hu et al., 2022). PGD2 has anti-inflammatory and immunomodulatory functions and plays important roles in regulating the immune responses and maintaining immune homeostasis (Xu et al., 2024). Therefore, PTGDS may play a role in the immunomodulatory mechanism of vitiligo through its metabolite PGD2. In addition, the change in *PTGDS* expression may affect the normal functions of cells by regulating the oxidative stress level, which is related to the pathogenesis of vitiligo (Li et al., 2024). Monoacylglycerol lipase (MGLL) is a primary enzyme responsible for the hydrolysis of 2-arachidonoylglycerol (2-AG) for breaking down monoglycerides into glycerol and fatty acids (Gil-Ordóñez et al., 2018); it plays a crucial role in various pathological processes, including pain, inflammation, and oxidative stress (Fan et al., 2024). By hydrolyzing 2-AG, MGLL affects the levels of AA and its metabolites, which play important roles in immune regulation and inflammation, i.e., regulating T cell proliferation and cytokine secretion (Cisar et al., 2018; Deng and Li, 2020). Therefore, MGLL may participate in the immune response process of vitiligo by regulating the activation and functions of immune cells. PNPLA8 is a lipase 8 containing patatin-like phospholipase domains and is involved in regulating the production of fatty acids, such as AA, as well as regulation of melanin production and cell functions through activation of the PI3K/Akt/Gsk3β and MAPK signaling pathways (Tan et al., 2023; Mosca et al., 2021). In addition, PNPLA8 enhances the production of AA through various subclasses of phosphatidylethanolamine and phosphatidylcholine to increase the production of prostaglandin E2 (PGE 2), which is beneficial for cell growth (Ramanadham et al., 2015). Knockdown of PNPLA8 has been reported to increase lipid peroxidation and trigger the intrinsic apoptotic pathways (Moon et al., 2012). However, the specific roles of MGLL and PNPLA8 in vitiligo are not directly reported; thus, future studies may further explore the roles of these enzymes in vitiligo and their potential clinical applications. Our study also showed that the three key genes *PTGDS*, *PNPLA8*, and *MGLL* are biomarkers for predictive models of vitiligo. Although the diagnosis of vitiligo is relatively clear through clinical observations and the Wood’s lamp examination, AA and other unsaturated fatty acids can help determine the inflammatory and autoimmune statuses (Kuda et al., 2018; Schwarz et al., 2017).

We further conducted immune infiltration analysis of the key genes to understand the relationships between them and vitiligo as well as its pathological mechanism. This analysis showed that there were 19 different immune cells between the lesion and control groups and that the proportions of activated CD8 T cells, activated CD4 T cells, and effector memory CD4 T cells were higher in the lesion group. CD8 T lymphocytes play an important role in the pathogenesis of vitiligo and are responsible for the destruction of melanocytes (van den Boorn et al., 2009). These cytotoxic CD8 T cells are present in greater numbers in the blood of vitiligo patients compared to healthy controls and are associated with vitiligo activity (Strassner et al., 2017). CD4 T cell dysfunction is often observed in autoimmune diseases, and these cells also play an important role in the autoimmune pathogenesis of vitiligo. Early reports have shown that the CD4 and CD8 T cells in vitiligo primarily produce IFN-γ and TNF-α, which are characteristic of Th1/Tc1 cell polarization (Wańkowicz-Kalińska et al., 2003). Studies using transgenic mouse models of melanocyte-specific T cell receptors have shown that CD4 T cells are involved in skin decolorization (Lambe et al., 2006). In addition, our results show that *PTGDS*, *PNPLA8*, and *MGLL* are significantly associated with changes in multiple immune cells; here, *PTGDS* and *MGLL* have similar correlations with the immune cells, whereas *PNPLA8* has the opposite correlation of the other two genes. These results suggest that *PTGDS*, *PNPLA8*, and *MGLL* may be involved in the pathogenesis of vitiligo through immune responses.

The results of TF predictions show that 11 TFs could regulate the expression of *MGLL* and 4 TFs could regulate the expression of *PTGDS*. TFAP2A was identified as a common transcriptional regulator of the biomarkers PTGDS and MGLL; TFAP2A can bind to DNA and plays an important role in regulating cell proliferation, differentiation, and apoptosis. Several TFs, including TFAP2A, have been identified in the skin samples of psoriasis patients and have been associated with the development of skin lesions in psoriasis (Alena et al., 2017). The role of TFAP2A in vitiligo needs further exploration and experimental evidence. We also constructed a TF–miRNA–mRNA network, which is conducive to further exploration of the mechanisms of these biomarkers in the occurrence and development of vitiligo.

There are some limitations to our present study. First, although we verified the expression patterns of the key genes, the specific biological roles of the related genes in vitiligo have not been explored deeply. Future studies are thus needed for functional validation in combination with *in vivo* and *in vitro* models to confirm the roles of these genes in vitiligo. Second, given the small sample sizes and limited sources, the statistical power of the results presented herein may be inadequate. Therefore, increasing the sample size and adding multicenter samples can help improve the reliability and broader applicability of our findings. In addition, although the biomarkers identified herein show potential for the diagnosis of vitiligo, there may be limitations in distinguishing the different stages or subtypes of vitiligo. Future studies should thus consider including samples of patients with different subtypes and stages of vitiligo to better evaluate the application of the proposed approach in clinical typing.

In summary, our results suggest that AAM may be closely related to vitiligo and that the AAM-RGs can be used as diagnostic biomarkers of vitiligo. The different expression patterns of these genes can provide more information for the diagnosis and treatment of vitiligo. By analyzing the expression characteristics of these genes, we can better predict the responses of patients to different treatment schemes, provide a scientific basis for the development of personalized treatment regimens, and dynamically monitor the effects during treatment to improve the treatment effects as well as quality of life of the patients.

## Data Availability

The datasets presented in this study can be found in online repositories. The names of the repositories and accession numbers can be found in the article/Supplementary Material.

## References

[B1] Alena Z. Evgeny C. Mehta R. Baranova A. Tatarinova T. V. Bruskin S. (2017). Identification of transcriptional regulators of psoriasis from RNA-seq experiments. Methods Mol. Biol. 1613, 355–370. 10.1007/978-1-4939-7027-8_14 28849568

[B2] Chaaba R. Bouaziz A. Ben Amor A. Mnif W. Hammami M. Mehri S. (2023). Fatty acid profile and genetic variants of proteins involved in fatty acid metabolism could Be considered as disease predictor. Diagn. (Basel) 13 (5), 979. 10.3390/diagnostics13050979 PMC10001328 36900123

[B3] Cisar J. S. Weber O. D. Clapper J. R. Blankman J. L. Henry C. L. Simon G. M. (2018). Identification of ABX-1431, a selective inhibitor of monoacylglycerol lipase and clinical candidate for treatment of neurological disorders. J. Med. Chem. 61 (20), 9062–9084. 10.1021/acs.jmedchem.8b00951 30067909

[B4] Cunningham K. N. Rosmarin D. (2023). Vitiligo treatments: review of current therapeutic modalities and JAK inhibitors. Am. J. Clin. Dermatol 24 (2), 165–186. 10.1007/s40257-022-00752-6 36715849

[B5] Deng H. Li W. (2020). Monoacylglycerol lipase inhibitors: modulators for lipid metabolism in cancer malignancy, neurological and metabolic disorders. Acta Pharm. Sin. B 10 (4), 582–602. 10.1016/j.apsb.2019.10.006 32322464 PMC7161712

[B6] Duan H. Song W. Zhao J. Yan W. (2023). Polyunsaturated fatty acids (PUFAs): sources, digestion, absorption, application and their potential adjunctive effects on visual fatigue. Nutrients 15 (11), 2633. 10.3390/nu15112633 37299596 PMC10255902

[B7] Fan C. Du J. Yu Z. Wang J. Yao L. Ji Z. (2024). Inhibition of MAGL attenuates intervertebral disc degeneration by delaying nucleus pulposus senescence through STING. Int. Immunopharmacol. 20 (131), 111904. 10.1016/j.intimp.2024.111904 38518595

[B8] Friedman J. Hastie T. Tibshirani R. (2010). Regularization paths for generalized linear models via coordinate descent. J. Stat. Softw. 33 (1), 1–22. 10.18637/jss.v033.i01 20808728 PMC2929880

[B9] Gil-Ordóñez A. Martín-Fontecha M. Ortega-Gutiérrez S. López-Rodríguez M. L. (2018). Monoacylglycerol lipase (MAGL) as a promising therapeutic target. Biochem. Pharmacol. 157, 18–32. 10.1016/j.bcp.2018.07.036 30059673

[B10] Gu Z. Gu L. Eils R. Schlesner M. Brors B. (2014). Circlize Implements and enhances circular visualization in R. Bioinformatics 30 (19), 2811–2812. 10.1093/bioinformatics/btu393 24930139

[B11] Hänzelmann S. Castelo R. Guinney J. (2013). GSVA: gene set variation analysis for microarray and RNA-seq data. BMC Bioinforma. 16 (14), 7. 10.1186/1471-2105-14-7 PMC3618321 23323831

[B12] Hu S. Cai S. R. Y. Cai Y. Liu J. Han Y. Zhao Y. (2022). Glycoprotein PTGDS promotes tumorigenesis of diffuse large B-cell lymphoma by MYH9-mediated regulation of Wnt-β-catenin-STAT3 signaling. Cell Death Differ. 29 (3), 642–656. 10.1038/s41418-021-00880-2 34743203 PMC8901925

[B13] Kuda O. Rossmeisl M. Kopecky J. (2018). Omega-3 fatty acids and adipose tissue biology. Mol. Asp. Med. 64, 147–160. 10.1016/j.mam.2018.01.004 29329795

[B14] Lambe T. Leung J. C. H. Bouriez-Jones T. Silver K. Makinen K. Crockford T. L. (2006). CD4 T cell-dependent autoimmunity against a melanocyte neoantigen induces spontaneous vitiligo and depends upon Fas-Fas ligand interactions. J. Immunol. 177, 3055–3062. 10.4049/jimmunol.177.5.3055 16920942

[B15] Li W. Yao T. Zhang X. Weng X. (2024). Oxylipin profiling analyses reveal that ω-3 PUFA is more susceptible to lipid oxidation in sheep testis under oxidative stress. Anim. Reprod. Sci. 268, 107567. 10.1016/j.anireprosci.2024.107567 39068814

[B16] Liang Y. Lin F. Huang Y. (2022). Identification of biomarkers associated with diagnosis of osteoarthritis patients based on bioinformatics and machine learning. J. Immunol. Res. 13, 5600190. 10.1155/2022/5600190 PMC9208923 35733917

[B17] Lyu C. Sun Y. (2022). Immunometabolism in the pathogenesis of vitiligo. Front. Immunol. 10 (13), 1055958. 10.3389/fimmu.2022.1055958 PMC9684661 36439174

[B18] Moon S. H. Jenkins C. M. Kiebish M. A. Sims H. F. Mancuso D. J. Gross R. W. (2012). Genetic ablation of calcium-independent phospholipase A(2)γ (iPLA(2)γ) attenuates calcium-induced opening of the mitochondrial permeability transition pore and resultant cytochrome c release. J. Biol. Chem. 287, 29837–29850. 10.1074/jbc.M112.373654 22778252 PMC3436185

[B19] Mosca S. Cardinali G. Flori E. Briganti S. Bottillo I. Mileo A. M. (2021). The PI3K pathway induced by αMSH exerts a negative feedback on melanogenesis and contributes to the release of pigment. Pigment. Cell Melanoma Res. 34 (1), 72–88. 10.1111/pcmr.12910 32608114

[B20] Ni Q. Ye Z. Wang Y. Chen J. Zhang W. Ma C. (2020). Gut microbial dysbiosis and plasma metabolic profile in individuals with vitiligo. Front. Microbiol. 11, 592248. 10.3389/fmicb.2020.592248 33381090 PMC7768019

[B21] Post N. F. Ginski G. Peters R. Van Uden N. O. P. Bekkenk M. W. Wolkerstorfer A. (2023). Trained immunity in the pathogenesis of vitiligo. Pigment. Cell Melanoma Res. 36 (5), 348–354. 10.1111/pcmr.13101 37293969

[B22] Ramanadham S. Ali T. Ashley J. W. Bone R. N. Hancock W. D. Lei X. (2015). Calcium-independent phospholipases A 2 and their roles in biological processes and diseases. J. Lipid Res. 56 (9), 1643–1668. 10.1194/jlr.R058701 26023050 PMC4548770

[B23] Ritchie M. E. Phipson B. Wu D. Hu Y. Law C. W. Wei S. (2015). Limma powers differential expression analyses for RNA-sequencing and microarray studies. Nucleic Acids Res. 43 (7), e47. 10.1093/nar/gkv007 25605792 PMC4402510

[B24] Schwarz A. Bruhs A. Schwarz T. (2017). The short-chain fatty acid sodium butyrate functions as a regulator of the skin immune system. J. Invest. Dermatol 137, 855–864. 10.1016/j.jid.2016.11.014 27887954

[B25] Strassner J. P. Rashighi M. Ahmed R. M. Richmond J. M. Harris J. E. (2017). Suction blistering the lesional skin of vitiligo patients reveal useful biomarkers of disease activity. J. Am. Acad. Dermatol 76, 847–855. 10.1016/j.jaad.2016.12.021 28259440 PMC5392432

[B26] Tan Z. Deme P. Boyapati K. Claes B. S. R. Duivenvoorden A. A. M. Heeren R. M. A. (2023). Key regulator PNPLA8 drives phospholipid reprogramming induced proliferation and migration in triple-negative breast cancer. Breast Cancer Res. 25 (1), 148. 10.1186/s13058-023-01742-0 38017485 PMC10683240

[B27] Tanacan E. Atakan N. (2020). Higher incidence of metabolic syndrome components in vitiligo patients: a prospective cross-sectional study. Bras. Dermatol 95, 165–172. 10.1016/j.abd.2019.07.006 PMC7175042 32113676

[B28] Tu B. Fang R. Zhu Z. Chen G. Peng C. Ning R. (2023). Comprehensive analysis of arachidonic acid metabolism-related genes in diagnosis and synovial immune in osteoarthritis: based on bulk and single-cell RNA sequencing data. Inflamm. Res. 72 (5), 955–970. 10.1007/s00011-023-01720-4 36995411

[B29] Turolo S. Edefonti A. Mazzocchi A. Syren M. L. Morello W. Agostoni C. (2021). Role of arachidonic acid and its metabolites in the biological and clinical manifestations of idiopathic nephrotic syndrome. Int. J. Mol. Sci. 22 (11), 5452. 10.3390/ijms22115452 34064238 PMC8196840

[B30] van den Boorn J. G. Konijnenberg D. Dellemijn T. A. van der Veen J. P. W. Bos J. D. Melief C. J. M. (2009). Autoimmune destruction of skin melanocytes by perilesional T cells from vitiligo patients. J. Invest. Dermatol 129, 2220–2232. 10.1038/jid.2009.32 19242513

[B31] Wańkowicz-Kalińska A. van den Wijngaard RMJGJ Tigges B. J. Westerhof W. Ogg G. S. Cerundolo V. (2003). Immunopolarization of CD4+ and CD8+ T cells to Type-1-like is associated with melanocyte loss in human vitiligo. Lab. Investig. J. Tech. Methods Pathol. 83, 683–695. 10.1097/01.lab.0000069521.42488.1b 12746478

[B32] Wu T. Hu E. Xu S. Chen M. Guo P. Dai Z. (2021). clusterProfiler 4.0: a universal enrichment tool for interpreting omics data. Innov. (Camb) 2 (3), 100141. 10.1016/j.xinn.2021.100141 PMC8454663 34557778

[B33] Xu J. Xu Y. Hou L. He X. Li Y. Zhao J. (2024). Molecular basis of lipid and ligand regulation of prostaglandin receptor DP2. Proc. Natl. Acad. Sci. U. S. A. 121 (51), e2403304121. 10.1073/pnas.2403304121 39665758 PMC11665870

[B34] Ye Z. Chen J. Du P. Ni Q. Li B. Zhang Z. (2022). Metabolomics signature and potential application of serum polyunsaturated fatty acids metabolism in patients with vitiligo. Front. Immunol. 13, 839167. 10.3389/fimmu.2022.839167 35222431 PMC8866849

[B35] Ye Z. Shen Y. Jin K. Qiu J. Hu B. Jadhav R. R. (2021). Arachidonic acid regulated calcium signaling in T cells from patients with rheumatoid arthritis promotes synovial inflammation. Nat. Commun. 12, 907. 10.1038/s41467-021-21242-z 33568645 PMC7875984

[B36] Zhang Y. Liu Y. Sun J. Zhang W. Guo Z. Ma Q. (2023). Arachidonic acid metabolism in health and disease. MedComm (2020) 4 (5), e363. 10.1002/mco2.363 37746665 PMC10511835

